# Hypofractionated versus conventionally fractionated image-guided volumetric-modulated arc radiotherapy for localized prostate cancer: a phase II randomized trial from China

**DOI:** 10.18632/aging.202551

**Published:** 2021-02-26

**Authors:** Qiu-Zi Zhong, Xiu Xia, Hong Gao, Yong-Gang Xu, Ting Zhao, Qin-Hong Wu, Dan Wang, Hai-Lei Lin, Xiang-Yan Sha, Ming Liu, Gao-Feng Li

**Affiliations:** 1 Department of Radiation Oncology, Beijing Hospital, National Center of Gerontology, Institute of Geriatric Medicine, Chinese Academy of Medical Sciences, P. R. China

**Keywords:** prostate cancer, radiotherapy, IGRT, hypofractionated, toxicity, efficacy

## Abstract

Purpose: To determine the safety of hypofractionated imaging-guided (IG) volumetric-modulated arc radiotherapy (IG-VMAT; 70 Gy/28 fractions over 5.5 weeks) versus conventionally fractionated regimen (IG-VMAT; 80 Gy/40 fractions over 8 weeks) in Chinese patients with localized prostate cancer.

Method: In this randomized non-comparative phase II trial, 92 patients with localized prostate cancer were assigned to receive either hypofractionated IG-VMAT (HFRT; 70 Gy/2.5Gy/28f) or conventionally fractionated IG-VMAT (CFRT; 80 Gy/2Gy/40f). Primary endpoint was grade 2 or higher late gastrointestinal (GI) and genitourinary (GU) toxicity at 2 years. The GI/GU toxicity and biochemical relapse–free survival (bRFS) were compared between the two treatment groups.

Results: Median follow-up was 26 months. The incidence of grade 2 or higher late GI/GU toxicity was low in both groups; the 5-year cumulative incidence of Radiation Therapy Oncology Group grade 2 or higher GI/GU toxicity at 2 years was 7.6% with HFRT versus 10.3% with CFRT (*P* = 0.707). Biochemical control was not significantly different between the two groups; the 2-year bRFS was 94.6% for HFRT versus 95.0% for CFRT (*P* = 0.704).

Conclusion: Hypofractionated IG-VMAT appears to be equivalent to conventionally fractionated IG-VMAT in terms of toxicity in Chinese patients with localized prostate cancer.

## INTRODUCTION

Prostate cancer is the second most frequently diagnosed cancer and the fifth leading cause of cancer death in men worldwide [1, 2]. It is relatively common in Europe and the US, but is uncommon in Asia [3]. In China, the incidence of prostate cancer has been increasing due to the increase in life expectancy; the incidence rose from 4.62 cases per 100,000 men in 2000 to 21.62 cases per 100,000 men in 2004 [4, 5]. Because prostate-specific antigen (PSA) screening for early detection of prostate cancer is not routinely performed in China, patients with prostate cancer tend to be relatively older at initial diagnosis and to have adverse prognostic features such as advanced-stage disease and high Gleason score and PSA level [6–8].

External beam radiotherapy is a well-established curative modality for patients with localized prostate cancer [9–12]. Important progress in external beam radiotherapy over the past decade include induction with highly conformal doses to the clinical target volume (CTV) through intensity-modulated beams (intensity-modulated radiotherapy; IMRT) or volumetric arcs (volumetric-modulated arc therapy; VMAT) [13]; stereotactic body radiotherapy (SBRT) [14]; proton therapy [13]; and precise imaging guidance [15]. These advances have led to successful dose escalations and hypofractionated schedules, and significantly reduced the dose delivered to adjacent normal tissues such as the rectum and bladder, reduced side effects, and improved treatment outcomes in patients with localized prostate cancer [9–15].

In standard external beam radiotherapy, daily fractions of 1.8-2.0 Gy are delivered up to a total radical dose of 74-80 Gy over 7-8 weeks. Efficacy and toxicity of moderately hypofractionated radiotherapy (HFRT), delivered at 2.4-3.4 Gy per fraction over 19-26 fractions in 4-6 weeks, has been shown to be equivalent to that with conventionally fractionated radiotherapy (CFRT) [16–25]. The goal of HFRT is to reduce overall treatment time, without compromising outcomes; thus, it saves resources and decreases healthcare costs, and is also convenient for patients. Based on the evidence from previous landmark studies, the use of HFRT in patients with localized prostate cancer has increased in Western countries [26]; however, only a very small proportion of patients have received this treatment in China. The slow adoption of HFRT in China may be due to concerns about the toxicity in patients with a more advanced-stage disease that may require large CTV, or the lack of validation data in Asian populations. In addition, previous randomized controlled trials comparing the efficiency of HFRT and CFRT did not always include careful image-guided (IG) radiotherapy (IGRT) or used it during the latter stages of recently published Conventional or Hypofractionated High-Dose Intensity Modulated Radiotherapy for Prostate Cancer (CHHiP) trial [27]; other studies restricted enrollment of low-risk or intermediate-risk patients with a favorable prognosis [16–18, 22–25]. More recently, studies have focused on the toxicity of ultra-hypofractionated radiotherapy to further shorten the treatment duration for low-risk patients in the IGRT setting [28–30]. It should also be noted that the mean age of patients with prostate cancer at initial diagnosis is around 72 years in China, almost 5-7 years more than that of patients in the US and Europe [5, 6]. No studies have specifically examined the efficacy and safety of HFRT and CFRT in Asian populations.

We hypothesized that HFRT for localized prostate cancer would be as effective and safe as CFRT in Chinese populations in the modern radiotherapy era. In 2016, we initiated this randomized, pilot, phase II trial to determine whether a hypofractionated 5.5-week schedule of VMAT is as safe as a standard 8-week schedule in Chinese patients treated with IGRT.

## RESULTS

### Patient characteristics

**Figure 1:**
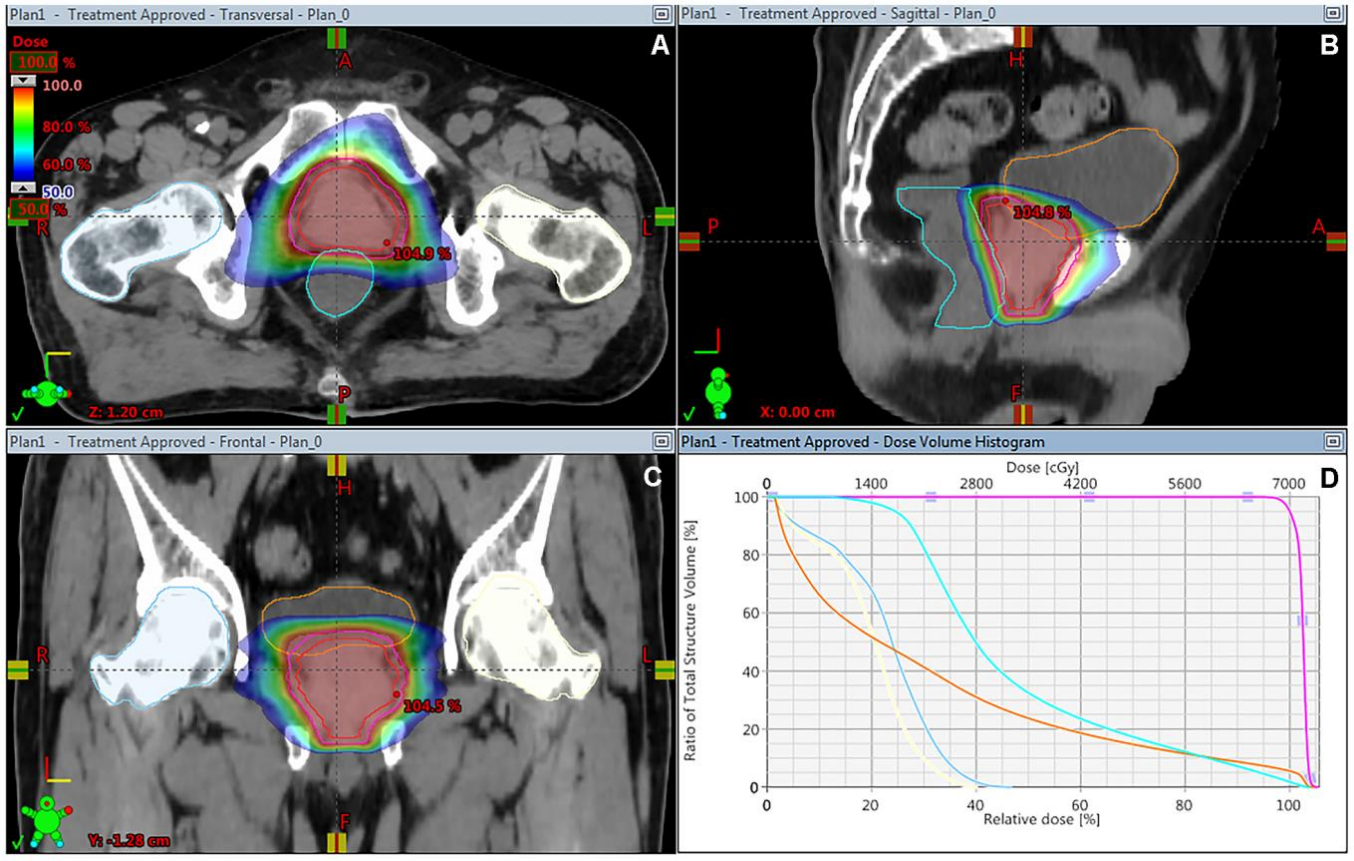
Dose distribution on the transverse (**A**), sagittal (**B**) and coronal (**C**) CT imaging, and DVH (**D**) of treatment planning of hypofractionated VMAT for patients with prostate cancer. DVH, dose–volume histogram; VMAT, volumetric modulated arc radiotherapy.

A total of 92 patients were enrolled between January 2016 and November 2018 and randomly assigned to either hypofractionated IG-VMAT (HFRT, 70 Gy/2.5 Gy/28 fractions; n = 46) or conventionally fractionated IG-VMAT (CFRT, 80 Gy/2 Gy/40 fractions; n = 46). All patients completed radiotherapy without interruption. Table 1 summarizes the clinical characteristics of patients in the two treatment arms. The median pre-therapy PSA level was 13 ng/mL. The dose coverage of PTV was not significantly different between the two groups (*P* = 0.639). Figure 1 shows a typical treatment planning of dose distribution and dose–volume histogram (DVH) with hypofractionated VMAT.

**Table 1:** Clinical characteristics and treatments among the two treatment groups.

	**HFRT**	**CFRT**	**Total**
	**n (%)**	**n (%)**	**n (%)**
Age (years)			
Range	54-84	61-86	54-96
≤ 70	4 (8.7)	9 (19.6)	13 (14.1)
> 70	42 (91.3)	37 (80.4)	79 (85.9)
Gleason score			
≤ 6	17 (37.0)	16 (34.8)	33 (35.9)
7	19 (41.3)	16 (34.8)	35 (38.0)
≥ 8	10 (21.7)	14 (30.4)	24 (26.1)
Initial PSA level (ng/ml)			
Mean	12.7	13.4	13.0
Median (range)	13 (5.8-41.7)	14 (4.9-54.2)	13 (4.9-54.2)
<10	12 (26.1)	14 (30.4)	26 (28.3)
10-20	24 (52.2)	20 (43.5)	44 (47.8)
>20	10 (21.7)	12 (26.1)	22 (23.9)
T stage			
T1	7 (15.2)	8 (17.4)	15 (16.3)
T2	25 (54.3)	26 (56.5)	51 (55.4)
T3	14 (30.4)	12 (26.1)	26 (28.3)
NCCN risk group			
Low risk	16 (34.8)	15 (32.6)	31 (33.7)
Intermediate risk	19 (41.3)	17 (37.0)	36 (39.1)
High risk	11 (23.9)	14 (30.4)	25 (27.2)

HFRT, hypofractionated radiotherapy; CFRT, conventionally fractionated radiotherapy; PSA, prostate-specific antigen; ADT, androgen-deprivation therapy.

NCCN, National Comprehensive Cancer Network.

### Acute toxicity

Median follow-up was 26 months (range, 3–39 months). Table 2 lists the acute and late GI and GU toxicities in the two groups. No patient developed grade 3–5 toxicities in either group during or after radiotherapy. Grade 1 acute toxicities occurred in 74/92 (80.4%) patients and grade 2 acute toxicities in 27/92 (29.3%) patients. The most common GI and GU toxicities were diarrhea and urinary frequency and urgency. The incidence of grade 1 and higher acute GI and GU toxicities was not significantly different between the two groups (*P* = 0.063).

**Table 2:** Comparison of acute and late gastrointestinal and genitourinary toxicities between the HFRT and CFRT groups.

**Toxicity**	**HFRT**	**CFRT**	
	**No (%)**	**No (%)**	**P**
**Acute toxicity**			
Genitourinary (GU)			0.132
Grade 0	16 (34.8)	20 (43.5)	
Grade 1	22 (47.8)	20 (43.5)	
Grade 2	8 (17.4)	6 (13.0)	
Gastrointestinal (GI)			0.190
Grade 0	21 (45.7)	26 (56.5)	
Grade 1	17 (37.0)	15 (32.6)	
Grade 2	8 (17.4)	5 (10.9)	
**Late toxicity**			
Genitourinary (GU)			0.496
Grade 0	40 (87.0)	39 (84.8)	
Grade 1	6 (13.0)	5 (10.9)	
Grade 2	0 (0.0)	2 (4.3)	
Gastrointestinal (GI)			0.915
Grade 0	40 (87.0)	41 (89.1)	
Grade 1	3 (6.5)	3 (6.5)	
Grade 2	3 (6.5)	2 (4.3)	

HFRT, hypofractionated radiotherapy; CFRT, conventionally fractionated radiotherapy.

### Late toxicity

**Figure 2:**
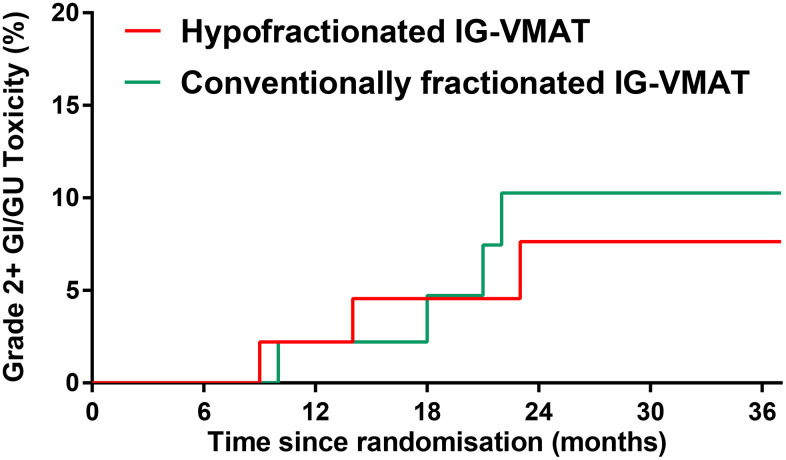
**RTOG grade 2 or higher late GI/GU toxicity in patients treated with hypofractionated IG-VMAT versus conventionally fractionated IG-VMAT.** RTOG, Radiation Therapy Oncology Group; GI, gastrointestinal; GU, genitourinary; IG, imaging-guided; volumetric-modulated arc therapy, VMAT.

The incidence of grade 2 and higher late GI/GU toxicity was low in both groups (Table 2). The 5-year cumulative incidence of RTOG grade 2 or higher GI/GU toxicity at 2 years was 7.6% in the HFRT group vs. 10.3% in the CFRT group (*P* = 0.707; Figure 2). The most common late radiation-related toxicity was mild (RTOG grade 0-1) rectal bleeding. No late grade 3 toxicity occurred.

### Disease-related outcome

**Figure 3:**
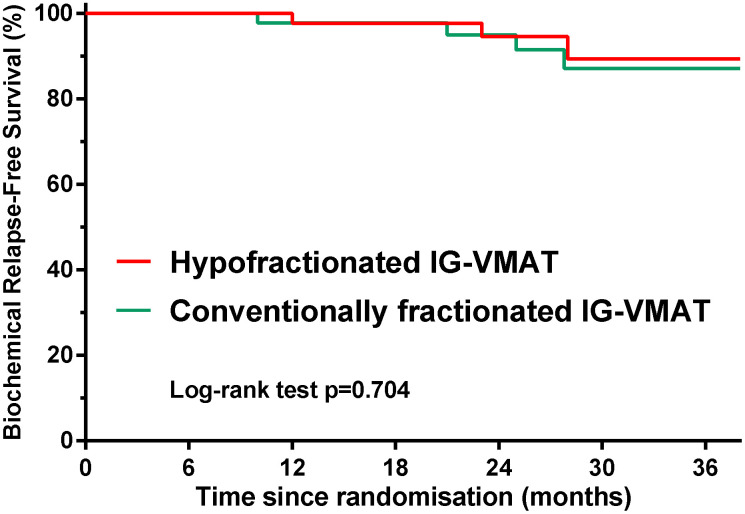
**bRFS outcome in patients treated with hypofractionated IG-VMAT versus conventionally fractionated IG-VMAT.** bRFS, biochemical relapse–free survival; IG, imaging-guided; volumetric-modulated arc therapy, VMAT.

Favorable prognosis was achieved after both HFRT and CFRT. In the HFRT group, 3/46 (6.5%) patients had PSA relapse. In the CFRT group, 2/46 (4.3%) patients had PSA relapse, and 1/46 (2.2%) patient developed distant metastasis. The difference in biochemical control was not significantly different between the two groups: the 2-year bRFS was 94.6% for HFRT vs. 95.0% for CFRT (*P* = 0.704, Figure 3). The median PSA nadir was 0.02 ng/mL in the CFRT group vs. 0.04 ng/mL in the HFRT group. The mean time to PSA nadir was 6.9 months in the CFRT group vs. 7.9 months in the HFRT group. No patient died from the disease in either group.

## DISCUSSION

This phase II randomized, non-comparative trial aimed to determine the toxicity profile of hypofractionated IG-VMAT in patients with localized prostate cancer in China. The risk of GI/GU toxicities was found to be low in patients treated with either hypofractionated VMAT or conventionally fractionated VMAT. The bRFS was also comparable with the two treatment regimens. Although this was a single-center study on a small sample, the findings support the use of hypofractionated IG-VMAT for treatment of localized prostate cancer in China.

External beam radiotherapy is a definitive treatment option for localized prostate cancer. Moderate hypofractionation is defined as radiotherapy with a fraction size between 2.4 Gy and 3.4 Gy [16–25]. The radiobiological rationale that a slow proliferation rate of prostate cancer is reflected by an α/β ratio of 1.5, similar to that of adjacent toxic effect–limiting OAR, suggests that hypofractionated radiation regimens will provide comparable disease control without increasing toxic effects. Several randomized controlled studies have compared moderate HFRT with CFRT for prostate cancer and demonstrated equivalent efficacy and toxicity with the two modalities [16–25]. The largest randomized trials to date included the CHHiP trial [20], the Dutch Hypofractionated versus Conventionally Fractionated Radiotherapy for Patients with Prostate Cancer (HYPRO) trial [21], the RTOG-0415 trial [22], and the Prostate Fractionated Irradiation Trial (PROFIT) [24]. RTOG-0415 randomized 1115 men with low-risk prostate cancer to receive either 73.8 Gy in 41 fractions of 1.8 Gy over 8.2 weeks or 70 Gy in 28 fractions of 2.5 Gy over 5.6 weeks. After median follow-up of 5.8 years, disease-free survival was found to be comparable between the two treatment groups [7]. IGRT was introduced only during the latter stages of the recently published CHHiP trial [27], and was shown to provide dosimetric benefit with minimal toxicity [15]. IMRT, often in the form of VMAT, achieves excellent target coverage, dose conformity, normal tissue sparing, and reduced treatment delivery time in prostate cancer. In the present study, in patients treated with hypofractionated IG-VMAT or conventionally fractionated IG-VMAT, we confirmed that the moderate fractionation dose of 2.5 Gy per fraction provides favorable biochemical control with low toxicity.

Improvements in imaging guidance and the adaptation of IMRT or VMAT have increased confidence in the precision of targeting of the prostate gland and the ability to minimize radiation to surrounding normal tissue. This has allowed successful dose escalation and increased interest in hypofractionation for treatment of prostate cancer. In the present study, overall toxicity in the hypofractionated IG-VMAT group was similar to that in the conventionally fractionated IG-VMAT group. Consistent with previous studies [15, 31, 32], we found the cumulative incidence of late GI/GU toxicity to be low in both fractionation groups in the IGRT setting. Previous randomized controlled trials have demonstrated equivalent toxicities with HFRT and CFRT in prostate cancer patients in the modern radiotherapy era [21, 24, 28, 29]. Only one study from RTOG-0415 found higher incidence of late grade 2 GI/GU toxicity in patients treated with HFRT, but this was probably due to the use of three-dimensional conformal radiotherapy in 21% of patients [22]. In studies with longer follow-up, no statistically significant difference in toxicity and quality of life have been observed between the two treatments [23].

One of the strengths of this study was that IG-VMAT was delivered with the latest radiotherapeutic techniques. In addition, the enrollment of a heterogeneous population makes the findings generalizable. Thus, the findings provide high-level evidence affirming that HFRT could be a practice standard for Chinese patients with localized prostate cancer [16–18, 22–25].

The main limitation of this trial was that the small sample size and the low frequency of events made efficacy analysis difficult; however, the results are entirely consistent with other trials [16–25]. Second, only bRFS was used to assess effectiveness of IG-VMAT. The data for bRFS or overall survival is not yet mature due to the short follow-up time. Despite these limitations, the results of this study can be helpful when designing a large multicenter phase III randomized trial of imaging-guided moderately hypofractionated VMAT versus conventionally fractionated VMAT in the modern radiotherapy era.

In conclusion, radiation-related toxicities and biochemical control appear to be favorable with both hypofractionated (70 Gy/2.5Gy/28f) and conventionally fractionated (80 Gy/2Gy/40f) IG-VMAT in Chinese patients with localized prostate cancer. The use of HFRT can benefit both the healthcare system and the patient by decreasing treatment costs and treatment time; these benefits can be particularly important in countries with limited resources.

## MATERIALS AND METHODS

### Patients and eligibility

This randomized non-comparative phase II trial recruited patients from a single academic hospital in China. Male patients were eligible if 1) they were aged ≥50 years, 2) had histologically confirmed prostate adenocarcinoma, 3) had good performance status (Eastern Cooperative Oncology Group [ECOG] score 0-1), and 4) had clinical stage T1-3 disease by the 2009 American Joint Committee on Cancer (AJCC) criteria. Exclusion criteria were 1) clinical stage T4, 2) evidence of nodal or distant metastases, 3) previous pelvic radiation therapy, or 4) previous malignancies. The clinical stage work-up included physical examination; biochemistry; PSA; computed tomography (CT) scans of the chest, abdomen, and pelvis; magnetic resonance imaging (MRI) of the pelvis and prostate; and whole-body bone scan.

Patients were categorized as low-risk, intermediate-risk, or high-risk according to the National Comprehensive Cancer Network (NCCN) guidelines [33]. Low-risk patients had cT1c–T2aN0M0, Gleason score ≤6, and PSA <10 ng/mL. Intermediate-risk patients had at least one of the following risk factors: T2c, Gleason score 7, or PSA 10-20 ng/mL. High-risk patients had at least one of the following risk factors: stage T3a or T3b, Gleason score ≥8, or PSA >20 ng/mL.

### Study design and treatment regimens

A computer-generated central randomization schedule was used to assign eligible patients in a 1:1 ratio to receive either conventionally fractionated IG-VMAT at 80 Gy in 40 fractions (2.0 Gy/fraction) over 8 weeks (CFRT group) or hypofractionated IG-VMAT at 70 Gy in 28 fractions (2.5 Gy/fraction) over 5.6 weeks (HFRT group), The total dose of 70 Gy in 28 fractions is biologically equivalent to 80 Gy in 40 fractions according to the α/β ratio of 1.5 Gy for prostate cancer.

As per NCCN guidelines [33], intermediate-risk and high-risk patients received, respectively, 4-6 months and 24 months of neoadjuvant/concurrent androgen deprivation therapy.

### Ethical compliance

All procedures were in conformance with the Helsinki declaration. The study protocol was approved by the local ethics committee and was registered at http://clinicaltrials.gov/ (no. NCT02934685). All patients were explained and signed written informed consent at enrollment.

### Radiotherapy technique

All patients underwent CT simulation with 3-mm slice thickness. Patients were instructed to have a full bladder and empty rectum at CT simulation and during the period of irradiation. The CTV included the whole prostate with or without seminal vesicles, depending on whether the patient was classified as high-, intermediate- or low- risk by NCCN criteria. The planning target volume (PTV) was the CTV plus a surrounding 5-mm margin—except posteriorly, where only a 3-mm margin was included. The bladder, rectum, small bowel, and femoral heads were delineated as organs at risk (OAR). The rectum volume included the entire rectal wall and lumen from the anus to the rectosigmoid flexure. The bladder was contoured from its base to the dome.

IG-VMAT was delivered without placement of fiducial markers. All doses were prescribed to a minimum isodose line encompassing 95% of the PTV. Planning CT and the verification cone-beam computed tomography (CBCT) were registered first with automatic bony registration, followed by a manual registration based on the soft-tissue alignment and prostate position in the CBCT. CBCT was performed daily in the HFRT group whereas, in the CFRT group, CBCT was performed daily during the first five fractions and, thereafter, three times a week.

Dose constraints for the HFRT group were as follows: volume of bladder receiving 25 Gy and 50 Gy were <65% and <55%, respectively, and volume of rectum receiving 25 Gy and 50 Gy were <60% Gy and <50%, respectively. Dose constraints for the CFRT group were as follows: volume of bladder receiving 25 Gy and 50 Gy were <75% and <65%, respectively, and volume of rectum receiving 25 Gy and 50 Gy were <70% and <60%, respectively.

### Evaluation and endpoints

Assessment of toxicity was performed every week during treatment. Patients were followed up at 1 month after completion of chemotherapy, then every 3 months for 2 years, every 6 months for the next 5 years, and yearly thereafter.

The primary endpoint was acute and late toxicity. Additional endpoints included biochemical relapse–free survival (bRFS), which was calculated from the date of randomization to the date of biochemical (PSA) failure, disease progression, relapse, death, or last follow-up. The Phoenix definition was used for PSA relapse (absolute nadir plus 2 ng/mL). Acute gastrointestinal (GI) and genitourinary (GU) toxicities were evaluated according to the Common Terminology Criteria for Adverse Events (CTCAE) version 3.0. Late radiation toxicity was assessed using the Radiation Therapy Oncology Group (RTOG)/the European Organization for Research and Treatment of Cancer (EORTC) late radiation morbidity scale [34].

### Statistical analysis

This hypofractionated IG-VMAT study was non-comparative and powered to assess toxicity independently in each treatment technique group, using Simon single-stage design with exact p-values [35]. The primary endpoint was late RTOG GI/GU toxicity at 2 years from start of radiation therapy. The late RTOG grade ≥2 GI/GU toxicity-free rate at 2 years was set at 80%, with an expected rate of 92% [15]. With a 5% one-sided alpha and 80% power, 43 patients in the hypofractionated IG-VMAT group were required. Assuming that there would be a 5% non-compliance rate, we enrolled 46 patients per group. An equal number of patients in the conventionally fractionated IG-VMAT group was sought to obtain prospectively collected toxicity data for standard treatment, summing in a total of sample size of 92 patients.

The chi-square or Fisher exact test was used for testing relationships between variables. Time to first occurrence of late radiation-induced side effects was analyzed using the Kaplan–Meier method to calculate the cumulative proportion with events reported on 2-year assessment. The log-rank test was used to compare hypofractionated IG-VMAT and conventionally fractionated IG-VMAT. Statistical calculations were performed using SPSS 16.0 (SPSS Inc., Chicago, IL, USA).
